# Biomarker Detection of Lung Cancers Before Clinical Symptoms or the Use of Traditional Imaging Methods in the ITALUNG Trial

**DOI:** 10.3390/ijms27188376

**Published:** 2026-09-20

**Authors:** Simonetta Bisanzi, Valentina Russo, Laura Carrozzi, Cristina Sani, Giulia Picozzi, Alberto Utili, Mario Mascalchi, Francesca Maria Carozzi, Eugenio Paci, Marco Peluso, the ITALUNG Working Group

**Affiliations:** 1 Regional Laboratory of Cancer Prevention, Institute for Cancer Research, Prevention and Clinical Network (ISPRO), 50139 Florence, Italy; 2 Pulmonary Unit, Cardiothoracic and Vascular Department, University Hospital of Pisa, 56124 Pisa, Italy; 3 Clinical Epidemiology Unit, Institute for Cancer Research, Prevention and Clinical Network (ISPRO), 50139 Florence, Italy; 4 Department of Clinical and Experimental Biomedical Sciences “Mario Serio”, University of Florence, 50121 Florence, Italy

**Keywords:** lung cancer, LOH, MSI, cfDNA, screening

## Abstract

The ITALUNG trial (Italian Lung Cancer Screening Trial) is a randomised controlled trial that evaluated the efficacy of Low-Dose Computed Tomography (LDCT) for lung cancer screening of over 3100 high-risk individuals, smokers and ex-smokers, aged 55–69. ITALUNG was coordinated by the Institute for Cancer Prevention Research in Florence and carried out across Tuscany in three main screening centres: Florence, Pisa, and Pistoia. The ITALUNG study demonstrated lower long-term lung cancer (LC) and cardiovascular mortality in the screened groups, with notable benefits observed in women. When the performance of the ITALUNG biomarker panel, i.e., loss of heterozygosity and microsatellite instability (LOH/MSI) and circulating free DNA (cfDNA), was analysed in samples of blood and sputum from asymptomatic high-risk subjects screened for lung cancer, sensitivity (SE) was 90% at baseline screening. However, the respective roles of LDCT screening and fluid biomarkers in screening for lung cancer are not established, highlighting the need for biomarkers to be able to detect tumours not only at the time of diagnosis but also when tested in pre-diagnostic samples. Therefore, we evaluated the longitudinal changes in the performance of multiple biomarkers in the ITALUNG biomarker panel and/or its single components to identify LC patients in ITALUNG. The cohort consisted of 55 LC patients whose biological specimens were collected over several years before diagnosis. Good results were found in test specimens obtained close to the time of diagnosis; out of 21 LC cases, 20 were positive in the ITALUNG biomarker panel [95% SE; 95% C.I. 0.86–1.04], 18 for LOH/MSI (86% SE; 95% C.I. 0.71–1.01) and 17 for cfDNA (85% SE; 95% C.I. 0.70–1.00). The panel was able to identify over 80% of LC cases by analysing the pre-diagnostic samples collected within 3 years prior to diagnosis: out of six LC patients, five were positive in the ITALUNG biomarker panel (83% SE; 95% C.I. 0.53–1.13). In addition, over 67% of the early and resected cancer cases were detected by the multiple-biomarker panel when biomarker testing samples were obtained within 11 years prior to diagnosis. The positivity of the ITALUNG biomarker panel might be associated with both carcinogenic field effects and early tumour presence when using pre-diagnostic samples obtained several years before diagnosis.

## 1. Introduction

Lung cancer (LC) is the leading cause of cancer death worldwide and the third most common cancer diagnosis in men and women combined [1]. Across studies examining multiple treatment strategies, median overall survival was generally higher for chemoradiotherapy (15–45 months) compared with chemotherapy alone (6.0–15.6 months) [2]. Low-dose computed tomography (LDCT) is a radiological technique that may decrease the mortality rate of LC by allowing early tumour detection [3]. The US National Lung Screening Trial (NLST), the Dutch–Belgian Netherlands–Leuvens Longkanker Screening Onderzoek study, and other randomised trials, including the German Lung Cancer Screening Intervention study (LUSI) and the Italian Lung study (ITALUNG), demonstrated that LDCT screening may decrease LC-associated mortality [4]. However, the respective roles of LDCT screening and molecular biomarkers in screening and prevention strategies for LC are not established, highlighting the need for biomarkers to be able to detect tumours not only at the time of diagnosis but also when tested in pre-diagnostic samples.

To further improve LDCT screening and prevention programs, there is the need to identify molecular signatures capable of predicting lung cancer risk years before diagnosis, potentially opening the door to a new era of precision cancer prevention. Highly sensitive and minimally invasive biomarkers, possibly complementary to LDCT, are necessary to increase screening accuracy.

Since 1950, the role of tobacco smoking in LC aetiology has been confirmed by accumulating evidence [5], with approximately 80% of all LC cases attributable to tobacco smoke [6]. Cigarette smoke is a complex aerosol containing thousands of chemical compounds, some of them identified as carcinogens by the IARC [7], that can cause lung and other cancers by the induction of genetic changes and molecular alterations [8]. The occurrence of carcinogenic events associated with smoking prompted the investigation of several molecular biomarkers, including accumulated genetic damage, DNA methylation aberrations, loss of molecular heterozygosity (LOH), microsatellite instability (MSI), mutation insults, and microRNA and circulating free DNA (cfDNA) [9,10,11,12,13] in the body fluids of smokers, to identify biomarkers of risk for developing LC [14]. After a pilot biomarker study [15], we examined the formation of LOH/MSI, two tobacco smoking-associated genetic abnormalities [16,17], as well as the levels of cfDNA, a liquid biopsy biomarker of tumour burden [18,19], in asymptomatic at-risk participants in the ITALUNG randomised trial [13], to examine if multimodal screening might improve the screening efficiency at baseline. In that study, the ITALUNG biomarker panel showed good performance, with the identification of 94% of LC patients at baseline LDCT screening and 67% at annual repeat screening.

It is recognised that the best combination of LDCT and molecular biomarkers for LC screening has yet to be determined [20,21], highlighting the need for biomarkers, amongst other factors, to be able to distinguish between LC cases and controls, not only at the time of diagnosis but also when tested using pre-diagnostic specimens [22]. Hence, we examined the longitudinal changes in the performance of multiple biomarkers in the ITALUNG biomarker panel and/or of its single components, i.e., LOH/MSI and cfDNA, to detect lung tumours in plasma samples, which were taken several years prior to diagnosis. The main aim of the study was to assess whether molecular biomarkers may be used for early LC diagnosis before clinical symptoms or the use of traditional imaging methods, thereby lowering the disease’s worldwide burden.

## 2. Results

### 2.1. Patient Characteristics

At the end of the 11-year follow-up, 91 LC cases occurred in the active group, with 55 patients previously analysed with regard to biomarker status [13]. Among the 55 LC cases that were previously analysed, 36 had screen-detected LC and 19 had cancers clinically presenting after the last screening LDCT examination or had interval cancers.

### 2.2. Performance of the ITALUNG Biomarker Panel and Its Components According to Sampling Time at Baseline to the Time of Diagnosis of Lung Cancer

Table 1 reports the number, SE and 95% C.I. of positive cases in multiple biomarker testing, specifically LOH/MSI and cfDNA, after stratification by sampling time to the diagnosis of LC. The ability of the biomarkers to correctly identify LC cases was then longitudinally analysed, considering five time periods since sampling: [0–1 year], [1–2 years], [2–3 years], [3–7 years] and [7–11 years]. The best performances were obtained when biomarkers were used for testing biological specimens collected within one year prior to diagnosis. In detail, out of 21 LC cases, 20 were positive in the multiple biomarker test (95% SE; C.I. 0.86–1.04), 18 for LOH/MSI (86% SE; C.I. 0.71–1.01) and 17 for cfDNA (85% SE; 0.70–1.00) (Table 1), showing that the sensitivity of the multiple-biomarker panel was higher than that of individual components. Results indicated that the multiple-biomarker panel was also able to identify over 80% of the LC cases when pre-diagnostic samples taken within 3 years prior to diagnosis were examined.

### 2.3. Performance of the ITALUNG Biomarker Panel According to Detection Modality and Tumour Characteristics

Table 2 reports the number, SE and 95% C.I. of positive cases in the panel after stratification by detection modality and tumour characteristics. According to cancer stage and tumour resectability, over 83% of the LC patients that were screen-detected at baseline had a positive result in the test, whereas lower estimates were found in the subsets of LC patients that were screen-detected at repeated rounds as well as for interval and post-trial cancers. When the histology at diagnosis was examined, the panel was found to be effective in the detection of over 71% of adenocarcinomas.

### 2.4. Performance of the ITALUNG Biomarker Panel According to Sampling Time at Baseline to the Time of Diagnosis of Lung Cancer, Stratified for Detection Modality and Tumour Characteristics

Table 3 shows the number and frequency of LC cases who tested positive in the ITALUNG biomarker panel after stratification by sampling time to the diagnosis of LC, according to detection modality and tumour characteristics. When the longitudinal changes in the performance of the multiple-biomarker panel were examined considering detection modality, over 95% of the screen-detected and interval and post-trial cases were identified through the testing of biological specimens that were collected close to the time of diagnosis (Table 3). Indeed, out of 20 patients with screen-detected LC, 19 had positive results (95% SE; 95% C.I. 0.85–1.05) within 1 year prior to diagnosis. Good performance was also found for cases with early-stage and resectable cancer (100% SE; 95% C.I. 1.00–1.00) and for those with advanced-stage and unresectable cancer (88% SE; 95% C.I. 0.65–1.11). In addition, 100% of adenocarcinomas and the squamous cell carcinomas were detected by the panel when samples were tested in this timeframe. The performance remained elevated when testing pre-diagnostic samples obtained within 3 years prior to diagnosis; over 83% of the screen-detected and early resected cancers showed a positive result in this time interval.

## 3. Discussion

LC screening programs in western countries are targeting individuals over the age of 50 with smoking histories [23]; the low frequency of LC in the target population should not prevent at-risk subjects from being directed toward cancer prevention programs [24]. Identifying molecular events preceding malignant transformation could aid risk stratification and prevention strategies. The ITALUNG trial is a randomised controlled trial that evaluated the efficacy of Low-Dose CT (LDCT) for early LC detection in high-risk smokers. The ITALUNG trial was a major Italian randomised controlled trial that investigated the efficacy of LDCT to screen for LC in smokers and ex-smokers aged 55 to 69. ITALUNG was conducted primarily in Tuscany, including centres in Florence, Pisa, and Pistoia, and showed that LDCT screening may reduce LC and overall mortality [25,26,27].

The trial showed a meaningful reduction in LC mortality, with studies indicating a significant drop (up to 24–30% in extended follow-up datasets) for those receiving LDCT screening. Long-term data also indicated a more pronounced mortality benefit in women compared to men. Conversely, the ITALUNG biomarker study expanded into multimodal screening approaches, collecting and storing blood and sputum samples in a biobank to test whether molecular biomarkers can improve the sensitivity and specificity of LDCT. When the performance of the ITALUNG biomarker panel, i.e., loss of heterozygosity and microsatellite instability (LOH/MSI) as well as circulating free DNA (cfDNA), was analysed in samples of blood and sputum of asymptomatic high-risk subjects screened for LC [13], SE was 90% at baseline screening, while specificity was 71% and 61% for LDCT and the ITALUNG biomarker panel as a baseline single test, respectively. Specificity improved to 89% with multimodal, combined screening.

Although some promising candidate biomarkers have been reported over the last few years, no efficient molecular biomarker is currently available in clinical practice for early identification of LC patients [28]. A typical criticism is that while molecular biomarkers are able to distinguish between symptomatic LC cases and controls at the time of diagnosis, they are not able to do so in pre-diagnostic samples [22]. To increase LDCT screening accuracy, it would be necessary to have more information with regard to how the longitudinal changes in biomarker performances are linked to the diagnosis of LC. The existence of a timeframe for biomarker testing that is efficient for the early detection of LC would represent an opportunity for increasing screening accuracy.

Although the study concentrated mostly on the 55 LC patients, the findings showed significant SE for the ITALUNG biomarker panel in the LC patients, including pre-diagnostic samples, reflecting both non-specific smoking-related airway damage and changes unique to tumours. Positive results 3 to 11 years prior to the diagnosis suggest ITALUNG results might be associated with increased baseline cancer risk as well as carcinogenic field effects in heavy smokers.

Our study shows that the ITALUNG biomarkers allowed accurate identification of LC cases when biological samples, acquired close to the time of the diagnosis, were examined (95% SE; 95% C.I. 0.85–1.04). The discriminatory ability of the test remained elevated when testing pre-diagnostic samples taken within 3 years prior to diagnosis, where over 80% of the cases yielded a positive result in this timeframe. The ITALUNG biomarker panel was found to be effective for the identification of LC after stratification for detection modality and tumour characteristics: over 83% of the subjects with LC that were screen-detected at baseline had positive biomarker testing, whereas lower SE estimates were computed for the subsets of the patients that had LC that were screen-detected at repeated rounds or involved interval and post-trial cancers. When the longitudinal changes in test performance were considered, over 88% of the LC patients whose biological samples were collected close to the time of diagnosis had a positive result. More than 83% of the screen-detected and the early-detected cases had positive biomarker testing when pre-diagnostic samples, obtained within 3 years prior to diagnosis, were examined. Over 67% of the early and resected cancer patients were identified using biological samples obtained within 11 years prior to diagnosis.

Next, the ITALUNG findings suggest a potential relationship between the presence of malignant lung nodules and positive biomarker testing, which may be of particular importance to evaluate the probability of nodule malignancy. To the best of our knowledge, the sensitivity reached by our multiple-biomarker panel was higher than that of LDCT, which ranged from 59% to 100% in a meta-analysis of 13 studies [29]. In our recent study [30], we demonstrated that increased baseline cfDNA may help to differentiate subjects with malignant and benign nodules at LDCT in ITALUNG, using the same cut-off of 3.15 ng cfDNA/mL plasma applied in these analyses.

As mentioned, positive results 3 to 11 years prior to the diagnosis point in ITALUNG might reflect both early tumour presence and carcinogenic field effects in heavy smokers. For instance, Smith at al. [31] showed that the sites most strongly involved in the formation of genetic lesions caused by tobacco smoking exposure were those with the highest mutation frequency in human LC. While the lung cancerogenic process may induce molecular alterations in surrogate tissues [32], the positivity of the LOH/MSI component may reflect progressive accumulation of genetic and molecular alterations during LC development [33]; normal tissue is replaced, changing from apparently phenotypically normal pre-neoplastic cells to cancer. Further hypotheses imply that test positivity may reflect the occurrence of unfavourable combinations of genetic traits that augment the effects of tobacco smoking-associated insults and confer increased risk of LC in susceptible individuals [34].

A number of initial developmental investigations conducted in this field have shown promising results [9,10,11,35,36,37,38,39]. Wang et al. [40] showed the detection of cancers by cfDNA in samples collected 3.1 to 3.5 years prior to clinical diagnosis. Multiple plasma proteins were also linked to lung cancer more than 5 years before diagnosis in the Canakinumab Anti-inflammatory Thrombosis Outcome Study [39]. In that trial, the 14-protein plasma signature was increased in both current smokers and individuals exposed to particulate matter and linked to lung myeloid and alveolar cells using a machine learning approach. In another investigation [9], *PTGER4*, *RASSF1A*, and *SHOX2* methylation alterations combined with radiological features were employed to build machine learning-based models aimed to distinguish malignant from benign pulmonary NCNs. Analysis of *SHOX2*/*PTGER4* methylation aberration in the body fluids of LC cases demonstrated high diagnostic performance [37]. A combination of liquid biopsy and *SHOX2*/*PTGER4* DNA methylation was developed for identifying LC at early stages [41]. Recently, a plasmatic lipidomic signature was found to be dysregulated in patients with early-stage LC [10]. Using different Artificial Intelligence networks, plasmatic proteins were predictive of LC [35]. A predictive model for personalizing screening intervals based on the combined use of LDCT and blood microRNA at baseline was also developed by Pastorino et al. [11]. Finally, host genetic susceptibility as measured by micronucleus was proven to be useful for the identification of susceptible persons who may be eligible for LDCT screening [36].

The presence of common molecular biomarkers in LC patients years before clinical symptoms suggests a strong link to cancer development and tobacco smoking exposure, providing an opportunity to improve screening strategies and to understand early carcinogenic processes. Improved performance of the ITALUNG biomarker panel is expected for the biological specimens of LC cases taken close to the time of diagnosis. With increased time prior to diagnosis, the proportion of subjects that are positive for ITALUNG biomarkers might decrease, ultimately becoming comparable to the rates of those without cancer. However, a limitation of this study is that adjustments for confounders, such as smoking intensity, chronic obstructive pulmonary disease (COPD), age, sex differences and comorbidities, which could influence the levels of cfDNA and LOH/MSI, were not performed. Also, the very small sample sizes in several subgroups should be considered when interpreting the ITALUNG findings. The methodology adopted in this study, both for data storage and sample analysis, was based on the technologies available at the beginning of the study [42], and the analytical results [13] have not yet been reproduced using more recent technological innovations. Research work is in progress to confirm the results in the biobank and validate the analytical process in more recent ongoing studies. A roadmap for revalidating/transposing the ITALUNG biomarker panel in light of currently available technologies should be based on the use of platforms and standards currently available in oncology diagnostics and research, such as whole-transcriptome and molecular karyotype measurements using next-generation human transcriptomes (Affymetrix HTA2.0) [43] and mutational profiles of LC-related gene analysis using platforms like Illumina MiSeq and IonS5 (Thermo Fisher, Waltham, MA, USA) or whole-genome sequencing [44] together with cfDNA analysis using PCR-based assays. This would allow us to transform what might appear to be a technological limitation into a valuable asset.

## 4. Materials and Methods

### 4.1. Study Design

ITALUNG is a randomised clinical trial based on a selection of at-risk persons aged 55–69 years, with a tobacco smoking history of at least 20 pack-years in the last 10 years. Study design, enrolment procedure and characteristics of the cohorts were previously reported [13]. In brief, letters were sent to residents in three districts of the Tuscany Region of Italy. Subjects were randomised to the screening intervention or the usual care (no screening) arms. The intervention arm (*n* = 1613) was invited to attend a screening cycle, consisting of a baseline and three annual repeat LDCT examinations. A total of 1406 asymptomatic at-risk subjects were screened at baseline and participated in the screening cycle. In the presence of solid lung nodules ≥ 5 mm at baseline or new solid lung nodules ≥ 3 mm at repeated rounds, the study provided recall for further assessment with diagnostic work-up, in accordance with a strict protocol [45]. Out of 1406 screened, 1356 subjects provided their individual consent to provide a sample of blood and sputum at baseline for biobank storage and molecular analyses; a second sample was requested when subjects were recalled for further assessment after baseline or at annual repeat rounds. Specificity, false-positive rates, positive predictive value and negative predictive value were previously estimated using matched non-cancer controls from the ITALUNG biobank across the same time periods [13,26]. Study procedures were performed in agreement with the Declaration of Helsinki for human studies and the guidelines of the Local Ethics Committee of each participating institution, who reviewed and approved the present investigation: Florence General Hospital approval number 29-30 of 30 September 2003; Pistoia General Hospital number 23 of 27 October 2003; and Pisa General Hospital number 00028543 of 13 May 2004. The study is registered at ClinicalTrial.gov (ID5NCT02777996).

### 4.2. Circulating Free DNA, Loss of Heterozygosity and Microsatellite Instability

The definition of a positive LOH/MSI result was defined and tested in a previous pilot study [15], whereas the cfDNA cut-off of 3.15 ng/mL was based on a recent re-analysis of the ITALUNG biomarker cfDNA data [30]. In detail, plasmatic levels of cfDNA were quantified by qRT-PCR [13] using the cut-off of 3.15 ng cfDNA/mL plasma, which better distinguished malignant and benign lung nodules in a recent study [30]. LOH/MSI components in plasma and sputum samples were analysed as previously reported [13]. A subject was considered to have a positive result for the LOH/MSI component when one sample (plasma or sputum) was found to be positive.

### 4.3. Statistical Analysis

The study population was grouped by sampling time with respect to diagnosis of LC until the end of the follow-up, which was extended to 11 years [46]. For this purpose, five time intervals were defined from the first sampling to the time of diagnosis: [0–1 year], [1–2 years], [2–3 years], [3–7 years] and [7–11 years]. The starting time was clearly defined as the sampling time at baseline, whereas the time-to-event of interest was the time between sampling and diagnosis of LC. The positivity of LOH/MSI at [0–1], [1–2] and [2–3] time intervals was designed around key screening rounds of the ITALUNG trial: a baseline LDCT scan at year 0, followed by three annual rounds at years 1, 2 and 3. Whereas, the [3–7] timeline reflected the first report, including the compensatory drop, and [7–11] represented the after-screening follow-up. The cohort was then stratified by detection modality, staging, surgical resectability and tumour histology. Standard descriptive statistical analyses were used to calculate the positivity rates of LC patients in the multiple-biomarker panel and/or its single components, i.e., LOH/MSI and cfDNA, along the different time intervals. Sensitivity (SE) was measured within each specific subgroup separately, as previously reported [47], to calculate the ability of the ITALUNG test and/or its single components to correctly identify LC cases. The denominator used for SE calculation was the total number of ITALUNG biomarker panel positive LC cases in the sample, calculated as True Positive (TP) LC patients plus False Negative (FN) LC patients. The formula for the calculation of sensitivity was SE = TP/TP + FN. Data were analysed using IBM SPSS Statistics^®^ software (version 20.0, Chicago, IL, USA).

## 5. Conclusions

The positivity of the ITALUNG biomarker panel might reflect both carcinogenic field effects and early tumour presence using pre-diagnostic samples obtained at different time points before clinical symptoms or the use of traditional imaging methods. Although promising, the use of multiple molecular biomarkers for early diagnosis of LC in screening and standard of care settings and for the management of pulmonary nodules is premature. Before determining that a multimodal screening approach integrating biomarkers and LDCT might improve screening programs, there is a need for external validation, larger sample size, other parameters such as specificity and positive predictive value estimates, and comparison with other existing biomarkers. Currently, we suggest two strategies for validating our data in future studies using available screening datasets, possibly with prospective enrolment. One strategy consists of a validation study using larger LDCT screening cohorts. The other is an investigation of the potential of this multiple-biomarker panel to guide the management of patients with indeterminate pulmonary nodules and distinguish between benign and malignant nodules using an integrated Artificial Intelligence and molecular biomarker approach [43,47].

## Figures and Tables

**Table 1:** Longitudinal changes in the performance of the ITALUNG biomarker panel and its components, i.e., loss of heterozygosity and microsatellite instability (LOH/MSI) and circulating free DNA (cfDNA), used to identify lung cancer patients.

Performance of the Multiple Biomarkers and Single Components by Sampling Time to Diagnosis							
		ITALUNG Biomarker Panel		LOH/MSI		cfDNA ^a^	
Time Intervals	Lung Cancer Cases, *n*	*n* pos.; SE%	95% C.I.	*n* pos.; SE%	95% C.I.	*n* pos.; SE%	95% C.I.
[0–1 year]	21	20; 95%	0.86–1.04	18; 86%	0.71–1.01	17, (1); 85%	0.70–1.00
[1–2 years]	5	4; 80%	0.45–1.15	4; 80%	0.45–1.15	2, (2); 67%	0.26–1.08
[2–3 years]	6	5; 83%	0.53–1.13	5; 83%	0.53–1.13	2, (3); 67%	0.29–1.05
[3–7 years]	11	6; 55%	0.26–0.84	3; 27%	0.01–0.53	5; 45%	0.16–0.74
[7–11 years]	12	7; 58%	0.30–0.86	3; 25%	0.01–0.50	4, (2); 40%	0.12–0.68

^a^ Missing data in cfDNA samples are in brackets.

**Table 2:** Performance of the ITALUNG biomarker panel in identifying lung cancer patients by detection modality, considering staging classification and surgical resectability (**A**) and histological subtypes of tumours (**B**).

(**A**)									
**Performance of the ITALUNG biomarker panel by detection modality according to cancer stage and tumour resectability**									
	Screen-detected at baseline			Screen-detected at repeated rounds			Interval and post-trial cases		
	Lung cancer cases, *n*	*n* pos.; SE%	95% C.I.	Lung cancer cases, *n*	*n* pos.; SE%	95% C.I.	Lung cancer cases, *n*	*n* pos.; SE%	95% C.I.
Early and Resected	13	13; 100%	1.00–1.00	13	10; 77%	0.54–1.00	3	2; 67%	0.14–1.20
Unresected and Late	6	5; 83%	0.53–1.13	4	3; 75%	0.33–1.17	16	10; 63%	0.39–0.87
(**B**)									
**Performance of the ITALUNG biomarker panel by detection modality according to tumour histology**									
	Screen-detected at baseline			Screen-detected at repeated rounds ^a^			Interval and post-trial cases ^a^		
	Lung cancer cases, *n* (*n* = 19) ^b^	*n* pos.; SE%	95% C.I.	Lung cancer cases, *n* (*n* = 17) ^b^	*n* pos.; SE%	95% C.I.	Lung cancer cases, *n* (*n* = 19)	*n* pos.; SE%	95% C.I.
Adenocarcinoma ^c^	8	8; 100%	1.00–1.00	14	10; 71%	0.47–0.95	6	5; 83%	0.53–1.13
Squamous cell carcinomas	4	4; 100%	1.00–1.00	2	2; 100%	1.00–1.00	5	1; 20%	−0.15–0.55
Small cell carcinomas	2	1; 50%	−0.19–1.19	1	1; 100%	1.00–1.00	1	1; 100%	1.00–1.00
Carcinoid	2	1; 50%	−0.19–1.19	0			0		
Non-small cell lung cancer	1	1; 100%	1.00–1.00	0			1	1; 100%	1.00–1.00
Unclassified ^d^	2	2; 100%	1.00–1.00	0			6	3; 50%	0.10–0.90

^a^ Blank cells for certain intervals represent specific histology categories with no cases. ^b^ After review, a screen-detected case at repeated rounds was attributed at baseline. ^c^ Including bronchioloalveolar carcinoma. ^d^ The unclassified category refers to specific carcinomas that cannot be histologically classified more specifically as adenocarcinomas, squamous cell carcinomas, or adenosquamous carcinomas.

**Table 3:** Longitudinal changes in the performance of the ITALUNG biomarker panel in identifying lung cancer patients according to detection modality, staging and surgical resectability, and tumour histology.

**Performance of the multiple-biomarker panel by detection modality and cancer stage**												
Screen-detected ^a^				Interval and post-trial cases ^a^			Early and Resected ^a^			Late and Unresected ^a^		
Time intervals	Lung cancer cases, *n*	*n* pos.; SE%	95% C.I.	Lung cancer cases, *n*	*n* pos.; SE%	95% C.I.	Lung cancer cases, *n*	*n* pos.; SE%	95% C.I.	Lung cancer cases, *n*	*n* pos.; SE%	95% C.I.
[0–1 year]	20	19; 95%	0.85–1.05	1	1; 100%	1.00–1.00	13	13; 100%	1.00–1.00	8	7; 88%	0.65–1.11
[1–2 years]	4	3; 75%	0.33–1.17	1	1; 100%	1.00–1.00	2	1; 50%	−0.19–1.19	3	3; 100%	1.00–1.00
[2–3 years]	6	5; 83%	0.53–1.13	0			6	5; 83%	0.53–1.13	0		
[3–7 years]	6	4; 67%	0.29–1.05	5	2; 40%	−0.03–0.83	5	4; 80%	0.45–1.15	6	2; 33%	−0.05–0.71
[7–11 years]	0			12	7; 58%	0.30–0.86	3	2; 67%	0.14–1.20	9	5; 56%	0.24–0.88
**Performance of the multiple-biomarker panel by tumour histology**												
	Adenocarcinomas						Squamous cell carcinomas ^a^					
Time intervals	Lung cancer cases, *n*		*n* pos.; SE%		95% C.I.		Lung cancer cases, *n*		*n* pos.; SE%		95% C.I.	
[0–1 year]	9		9; 100%		1.00–1.00		4		4; 100%		1.00–1.00	
[1–2 years]	2		1; 50%		−0.19–1.19		2		2; 100%		1.00–1.00	
[2–3 years]	6		5; 83%		0.53–1.13		0					
[3–7 years]	6		4; 67%		0.29–1.05		2		0; 0%		0.00–0.00	
[7–11 years]	5		4; 80%		0.45–1.15		3		1; 33%		−0.20–0.86	

^a^ Blank cells for certain intervals represent specific histology categories with no cases.

## Data Availability

The original contributions presented in this study are included in the article. Further inquiries can be directed to the corresponding author.

## Appendix A

ITALUNG Working Group: Eugenio Paci, MD; Marco Zappa, MD; Francesca Maria Carozzi, BSc; Simonetta Bisanzi, BSc; Cristina Sani, BSc; Marco Peluso BSc; Jessica Viti, BSc; Giampaolo Pompeo, BSc; Regional Laboratory of Cancer Prevention, ISPRO, Florence, Italy; Mario Mascalchi, MD; Donella Puliti, Msc; Gianfranco Manneschi, MSc; Giulia Picozzi, MD; Jasmine Giovannoli, MSc; Clinical Epidemiology Unit, ISPRO, Florence, Italy; Andrea Martini, MSc; Environmental and Occupational Epidemiology Unit, ISPRO, Florence, Italy; Maurizio Bartolucci, MD; Department of Radiology, Azienda USL Toscana Centro, Santo Stefano Hospital, Prato, Italy; Massimo Falchini, MD; Interventional Radiology Unit Azienda USL Toscana Centro, Torre Galli Hospital Florence, Florence, Italy; Camilla Comin, MD: Pathology Department, Careggi Hospital, University of Florence, Florence, Italy; Luca Vaggelli, MD: Nuclear Medicine Department, Careggi Hospital, Florence, Italy; Laura Carrozzi, MD; Ferruccio Aquilini, BSc; Stella Cini, MD; Mariella De Santis, MSc; Francesco Pistelli, MD; Filomena Baliva, BSc; Antonio Chella, MD; Laura Tavanti, MD; Cardiothoracic and Vascular Department, University Hospital of Pisa, Pisa, Italy; Fabio Falaschi^‡^, MD; Annalisa De Liperi, MD; Cheti Spinelli, MD; Chiara Romei, MD; Radiology Department, University Hospital of Pisa, Pisa, Italy; Marco Lucchi, MD; Thoracic Surgery Unit, Cardiothoracic and Vascular Department, University Hospital of Pisa, Pisa, Italy; Gabriella Fontanini, MD; Greta Alì, MD; Adele Renza Tognetti, MD; Pathology Department, University Hospital of Pisa, Pisa, Italy; Michela Grazzini, MD; Ilaria Natali, BSc: Pneumonology Department, Hospital of Pistoia, Pistoia, Italy; Letizia Vannucchi, MD; Alessia Petruzzelli, MD; Anna Talina Neri, MD: Radiology Department, Hospital of Pistoia, Pistoia, Italy.
